# Superior predatory ability and abundance predicts potential ecological impact towards early-stage anurans by invasive ‘Killer Shrimp’ (*Dikerogammarus villosus*)

**DOI:** 10.1038/s41598-021-82630-5

**Published:** 2021-02-25

**Authors:** Daniel A. Warren, Stephanie J. Bradbeer, Alison M. Dunn

**Affiliations:** 1 School of Biology, University of Leeds, Leeds, LS2 9JT UK; 2 Water@Leeds, School of Geography, University of Leeds, Leeds, LS2 9JT UK

**Keywords:** Invasive species, Ecological modelling, Freshwater ecology, Herpetology

## Abstract

Invasive alien species negatively impact upon biodiversity and generate significant economic costs worldwide. Globally, amphibians have suffered considerable losses, with a key driver being predation by large invasive invertebrate and vertebrate predators. However, there is no research regarding the potential ecological impact of small invertebrate invaders. The invasive freshwater amphipod *Dikerogammarus villosus* can act as a top predator capable of displacing native amphipods and preying heavily upon a range of native species. Listed as one of Europe’s top 100 worst invaders, *D. villosus* has significantly restructured freshwater communities across western Europe and is expected to invade North America in the near future. Here we explore the ecological impact of invasive *D. villosus* upon UK native and invasive amphibians (*Rana temporaria* and *Xenopus laevis* respectively) using the “Relative Impact Potential” (RIP) metric. By combining estimations of *per capita* effects (i.e. functional response; FR) and relative field abundances, we apply the RIP metric to quantify the potential ecological impact of invasive *D. villosus* upon embryonic and larval amphibian prey, compared to the native amphipod *Gammarus pulex*. Both native and invasive amphipods consumed early-stage amphibians and exhibited potentially destabilising Type II FRs. However, larger body size in invasive *D. villosus* translated into a superior FR through significantly lower handling times and subsequently higher maximum feeding rates—up to seven times greater than native *G. pulex*. Higher invader abundance also drove elevated RIP scores for invasive *D. villosus*, with potential impact scores predicted up to 15.4 times greater than native *G. pulex*. Overall, *D. villosus* is predicted to have a greater predatory impact upon amphibian populations than *G. pulex*, due primarily to its larger body size and superior field abundance, potentially reducing amphibian recruitment within invaded regions.

## Introduction

Biological invasions are of increasing global concern, with invasive alien species (IAS) generating a substantial cost to the global economy, estimated to be more than $974 billion USD^1^. IAS can have an immense, often irreversible effect upon native communities and ecosystems, ranked second only to habitat destruction in terms of impact^2^. Freshwater ecosystems are spatially restricted (occupying ~ 0.8% of the Earth’s surface) yet highly biodiverse, supporting approximately 6% of all described species^3^. However, freshwaters experience a disproportionate incidence of IAS invasions^4^, with invader impacts typically more severe when compared to terrestrial ecosystems^5^. IAS influence native communities through a variety of trophic interactions, of which predation is key^6^. Compared to trophically analogous native species, invasive predators often consume prey at a higher rate (reviewed by Dick et al.^7, 8^; Cuthbert et al.^9^). Furthermore, IAS typically reach higher abundances in comparison to native analogues^8^, applying even greater predatory pressures upon local prey populations and assemblages.

Amphipod crustaceans (Order: Amphipoda) are frequently identified as high-impact freshwater invaders^10^. The ‘killer shrimp’ *Dikerogammarus villosus* (Sowinsky, 1894) is listed as one of the 100 worst invaders in Europe^11^, and is a species of high concern in Great Britain^12^, and North America^13^. *Dikerogammarus villosus* threatens freshwater biodiversity and ecosystem functioning throughout Western Europe, permanently altering the structure of invaded native assemblages across multiple trophic levels^14–16^. The invasive success of *D.* villosus is attributable to several life history characteristics, including a wide ecophysiological tolerance^17^, rapid growth and high fecundity^10, 18^, an effective anti-predator strategy^19^ and a strong competitive ability^20^. Acknowledged for its large body size, large mouthparts, flexible omnivory and superior predatory capabilities^21^, *D. villosus* is a voracious, high trophic predator^14^. In the laboratory, *D. villosus* readily consumes a wide range of freshwater macroinvertebrates (reviewed in Rewicz et al.^21^). This wide dietary range is also seen in the field, as confirmed by stable isotope analyses^14, 22^. Aquatic vertebrates may also be at risk, with reports of predation on fish eggs and larvae^23, 24^. However, to our knowledge there are no studies concerning the predatory impact of invasive *D. villosus* towards amphibians.

Regarded globally as a critical conservation concern, amphibians have experienced substantial declines over the past 40 years^25^. Current amphibian extinction rates are estimated to be four orders of magnitude greater than background extinction rates^26^, with approximately ~38% of known amphibian species threatened with imminent extinction^27^. Amphibian declines are driven by various factors, including climate change, environmental pollutants, habitat loss, pathogens (e.g. *Batrachochytrium dendrobatidis*^28^) and invasive species^29^. Of the ~7000 amphibian species listed on the International Union for Conservation of Nature (IUCN) Red List, 17% of species are directly threatened by invasive alien species, of which 11% of species are categorised as vulnerable, endangered or critically endangered^30–32^. Predation of embryonic and larval amphibians by large invasive freshwater predators, particularly fish, crayfish^33^, and other amphibians (e.g. bullfrogs) is one of the major contributors in the decline and extirpation of amphibian populations^34^. Whilst amphibians typically breed in ponds, lakes, streams, rivers and canals^35^, they can also occupy the same habitat as *D. villosus*, having previously been recorded in large invaded freshwater bodies in the UK^36^ (Anglian Water, pers. comm.; Cardiff Harbour Authority, pers. comm.), and also in mainland Europe^37–40^. Given that stable isotope analysis suggests that *D. villosus* can occupy the same trophic level as some predatory fish species^41^, this invasive amphipod may pose a potential risk to larval amphibians.

We present the first empirical study comparing the ecological impacts of invasive and native freshwater amphipod predators upon the early, aquatic life-stages of two amphibian species. The ecological impact of invasive predators is dependent on predatory capability, relative to native analogues, as well as relative abundance^8^. Here we compare the predatory functional responses of invasive *D. villosus* and native *Gammarus pulex* (Linnaeus, 1758) towards the embryos and larvae of native *Rana temporaria* (Linnaeus, 1758) (European Common Frog) and invasive *Xenopus laevis* (Daudin, 1802) (African Long-Clawed Frog). We also estimate relative abundances of native *G. pulex* and invasive *D. villosus* in field populations in Great Britain, and supplement these values using published estimations. We apply the Relative Impact Potential metric (see Dick et al.^8^), which incorporates relative consumer abundance as a means of scaling relative *per capita* effects to compare the relative impact potential of these freshwater amphipod species towards amphibians present in Great Britain.

## Results

### Predation of invasive *X. laevis* embryos

Prey survival was 100% in all control treatments, which was significantly higher than within invasive amphipod treatments (intermediate *D. villosus* = 84.8% and large *D. villosus* = 75.7% survival; Fisher’s exact test p < 0.001 for both), but not large *G. pulex* (99.4% survival; p = 0.06). Therefore, mortality was attributed to amphipod predation. When presented with invasive *X. laevis* embryos, predation by large native *G. pulex* was minimal, with only 5 of 45 individuals consuming embryos. By comparison, a significantly higher incidence of predation was observed in intermediate (i.e. size-matched with *G. pulex*) and large invasive *D. villosus* (44 of 45 individuals for both; χ^2^ = 105.138, df = 2, p < 0.001).

### Functional responses

**Figure 1:**
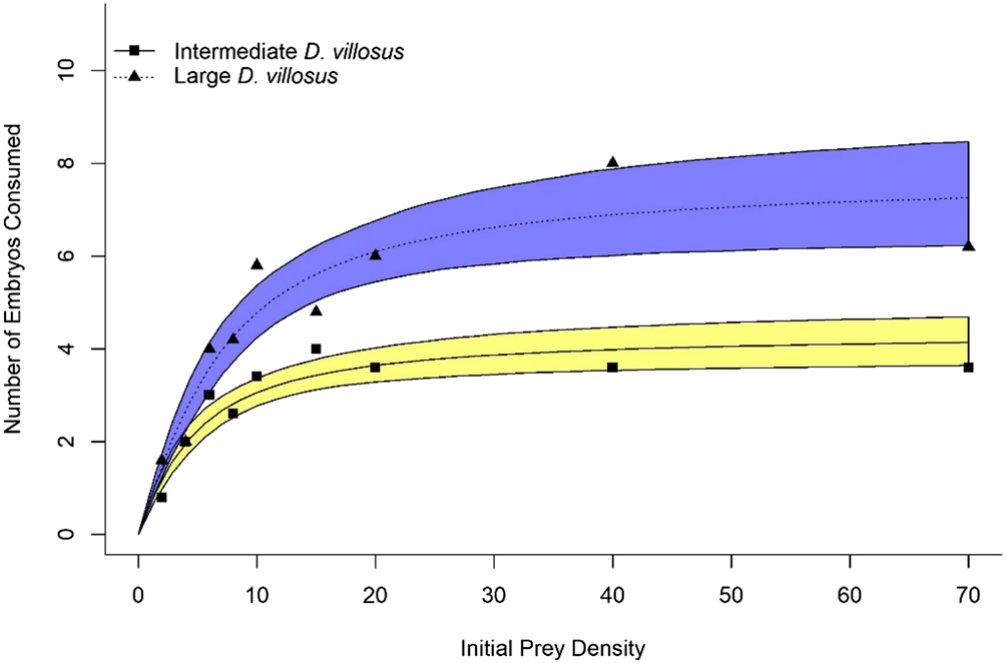
Type II functional response curves for intermediate *D. villosus* (filled squares and solid black line) and large *D. villosus* (filled triangle and dotted black line) towards non-native *X. laevis* embryos (n = 5 replicates per prey density). *Shaded Regions* are bootstrapped 95% confidence intervals (yellow: intermediate *D. villosus*, blue: large *D. villosus*).

Logistic regressions revealed significantly negative first order terms by invasive *D. villosus* against *X. laevis* embryos, confirming the expression of Type II FRs (Fig. 1; Supplementary Materials Table S6). When compared to intermediate *D. villosus*, large *D. villosus* displayed significantly lower handling times on *X. laevis* embryos, whilst attack coefficients (i.e. initial slope of FR curves; see Fig. 1) did not differ statistically (Tables 1, 2). Estimated maximum feeding rates (i.e. asymptote of FR curve; Fig. 1) were substantially higher in large *D. villosus*, consuming considerably more prey items during the experimental period, compared to intermediate invaders (Table 3). Negligible predation by large *G. pulex* prevented an FR curve from being plotted or compared with invasive amphipods. Significantly lower handling times and higher maximum feeding rates estimated for large *D. villosus* translated into a noticeably higher FR curve, compared to intermediate *D. villosus* (Fig. 1). Although 95% confidence intervals overlapped at lower prey densities, the expression of a steep initial FR gradient by larger individuals resulted in the separation of confidence intervals at higher densities.

**Table 1:** Functional response parameter estimates for each amphipod size group (intermediate *D. villosus*, large *D. villosus* and large *G. pulex*) feeding upon invasive *X. laevis* embryos and native *R. temporaria* larvae as prey. Estimates extracted from the Rogers’ Random Predator Equation, fitted in the *frair* package^97^.

Prey treatment	Amphipod group	Parameter	Estimate (± SE)	z	P
*X. laevis* embryos	Intermediate *D. villosus*	$a$	0.616 (± 0.186)	3.311	< 0.001***
		$h$	0.459 (± 0.061)	7.590	< 0.001***
	Large *D. villosus*	$a$	0.850 (± 0.183)	4.650	< 0.001***
		$h$	0.258 (± 0.028)	9.114	< 0.001***
*R. temporaria* larvae	Large *G. pulex*	$a$	0.120 (± 0.141)	0.853	0.394
		$h$	6.600 (± 2.721)	2.425	0.015*
	Intermediate *D. villosus*	$a$	0.597 (± 0.719)	0.529	0.407
		$h$	2.803 (± 0.709)	3.955	< 0.001***
	Large *D. villosus*	$a$	0.392 (± 0.150)	2.612	< 0.01**
		$h$	0.988 (± 0.213)	4.631	< 0.001***

$a$ – attack coefficient, $h$—handling time (days prey item^−1^), SE – Standard error.

Significant differences are indicated in bold. Asterisks indicate significance of P values; * = P < 0.05, ** = P < 0.01, and *** = P < 0.001.

**Table 2:** Comparison of functional response parameter estimates for three amphipod size groups (intermediate *D. villosus*, large *D. villosus* and large *G. pulex*) feeding upon invasive *X. laevis* embryos and native *R. temporaria* larvae as prey.

Prey treatment	Base group	Comparator group	Parameter	Estimate (± SE) of difference ($Da$ or $Dh$)	z	P
*X. laevis* embryos	Large *D. villosus*	Intermediate *D. villosus*	$a$	0.233 (0.262)	0.890	0.373
			$h$	− 0.202 (0.067)	− 3.016	**0.003**
*R. temporaria* larvae	Large *D. villosus*	Large *G. pulex*	$a$	0.272 (0.205)	1.328	0.184
			$h$	− 5.606 (2.726)	− 2.056	**0.040**
	Intermediate *D. villosus*	Large *G. pulex*	$a$	0.477 (0.731)	0.652	0.515
			$h$	− 3.795 (2.811)	− 1.350	0.177
	Large *D. villosus*	Intermediate *D. villosus*	$a$	− 0.204 (0.735)	− 0.278	0.781
			$h$	− 1.814 (0.740)	− 2.451	**0.014**

Comparisons based on analyse conducted using ‘indicator’ variables in the *frair* package^97^.

$a$ – attack coefficient, $h$—handling time (days prey item^−1^), SE – Standard Error.

Significant differences are indicated in bold.

**Table 3:** Mean Relative Impact Potential (RIP) scores, generated using mean ± standard error (SE) estimates of maximum feeding rate (FR) and field abundance (ind/m^2^), recorded for each amphipod group (intermediate *D. villosus*, large *D. villosus* and large *G. pulex*) whilst feeding upon native *R. temporaria* as prey.

Comparison		Predator A—mean FR parameter (± SE)	Predator B—mean FR parameter (± SE)	Predator A—mean field abundance (ind/m^2^ ± SE)	Predator B—mean field abundance (ind/m^2^ ± SE)	RIP	Uncertainty 80% CI 60% CI	${P}_{RIP}>1 (\%)$
Predator A (base group)	Predator B (comparator group)							
Large *D. villosus*	Large *G. pulex*	1.099 (± 0.047)	0.157 (± 0.012)	14.760 (± 2.955)	17.378 (± 4.486)	6.379	4.555 – 7.990 5.544 – 6.565	100
Intermediate *D. villosus*	Large *G. pulex*	0.469 (± 0.063)	0.157 (± 0.012)	83.280 (± 15.710)	17.378 (± 4.486)	15.359	10.748 – 19.404 13.213 – 15.784	100
Intermediate *D. villosus*	Large *D. villosus*	0.469 (± 0.063)	1.099 (± 0.047)	83.280 (± 15.710)	14.760 (± 2.955)	2.509	1.849 – 3.098 2.215 – 2.587	99.780

RIP scores are presented alongside estimates of uncertainty (60% and 80% confidence intervals; CIs) and the probability (%) that the RIP output will exceed 1. Mean (± SE) estimates of maximum feeding rates obtained through bootstrapping FR model n = 30.

### Predation of native *R. temporaria* embryos

Survivorship of native *R. temporaria* embryos within both control and experimental amphipod treatments was absolute. In 105 replicated trials (across the three amphipod treatments), no embryos were consumed, although evidence of attempted predation by invasive *D. villosus* was observed. As such, analysis could not be conducted further. In an additional trial to confirm whether *R. temporaria* embryos were palatable, amphipods were offered ten *R. temporaria* embryos which had been subjected to considerable mechanical damage (n = 3 replicates per amphipod group). Predation by invasive amphipods was observed, with large *D. villosus* consuming an average of 3.6 embryos and intermediate *D. villosus* consuming 2.3 embryos. *Gammarus pulex* did not consume any embryos.

### Predation of native *R. temporaria* larvae

Native *R. temporaria* larvae experienced negligible mortality in control treatments (1.2%), whilst mortality was significantly higher when exposed to large *D. villosus*, intermediate *D. villosus* and large *G. pulex* (18.8%, 9.5% and 3.6%, respectively; Fisher’s exact test p < 0.05 for all). Therefore, mortality was attributed to predation by amphipods. Predatory frequency was highest for large *D. villosus*, with 53 of 77 individuals (68.83%) consuming larvae. This was significantly higher than frequencies recorded in intermediate *D. villosus* (χ^2^ = 11.55, df = 1, p < 0.001), for which 31 of 77 individuals (40.26%) were observed consuming native *R. temporaria* larvae. Predation was significantly less frequent in large *G. pulex*, with only 16 of 76 individuals (21.05%) consuming larvae (p < 0.001 for both).

### Functional responses

**Figure 2:**
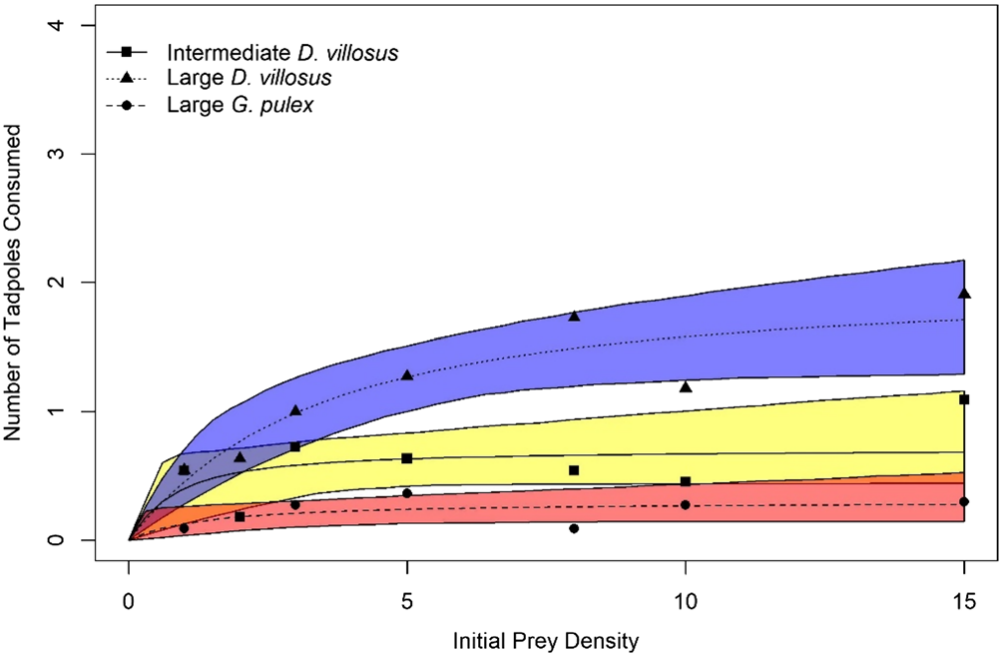
Rogers random-predator (Type II) functional response curves for large *G. pulex* (filled circles with dot-dash black line), intermediate *D. villosus* (filled squares and solid black line) and large *D. villosus* (filled triangle and dotted black line) towards native *R. temporaria* larvae (n = up to 11 replicates per prey density). *Shaded Regions* display bootstrapped 95% confidence intervals (red: large *G. pulex*, yellow: intermediate *D. villosus*, blue: large *D. villosus*).

Logistic regressions identified significantly negative first order terms in all amphipod groups (p < 0.05 for all), indicating that native and invasive amphipods expressed Type II FRs towards native *R. temporaria* larvae (Fig. 2; Supplementary materials Table S6). Estimates for attack coefficients were statistically similar between amphipod groups (p > 0.05; Tables 1, 2). Comparisons between size-matched native and invasive amphipods revealed non-significant differences in estimates of handling time. In contrast, handling times were significantly lower in large *D. villosus* when compared to intermediate *D. villosus* and large *G. pulex* (p < 0.05 for both). Maximum feeding rates estimated for large *D. villosus* were considerably higher than size-matched native and invasive amphipods (Table 3), up to seven times greater when compared to large *G. pulex*. Superior maximum feeding rates translated into a higher FR curve with a distinct separation from smaller amphipods (Fig. 2).

### Relative impact potential

Comparisons of total field abundance estimations revealed statistically significant differences in the abundances of native (mean ± SE, 170 ± 43.83 ind/m^2^) and invasive (870 ± 259.79 ind/m^2^) populations (GLM; F_(1,68)_ = 17.589, p < 0.001). When categorised based on body size, statistical comparisons indicated significant differences between the field abundance estimates for the three amphipod groups (F_(2,92)_ = 21.395, p < 0.001; Table 3). Post hoc analyses revealed that intermediate *D. villosus* were significantly more abundant when compared to large *D. villosus* and *G. pulex* (p < 0.001), whilst abundance estimates did not differ between *G. pulex* and large *D. villosus* (p = 0.925).

**Figure 3:**
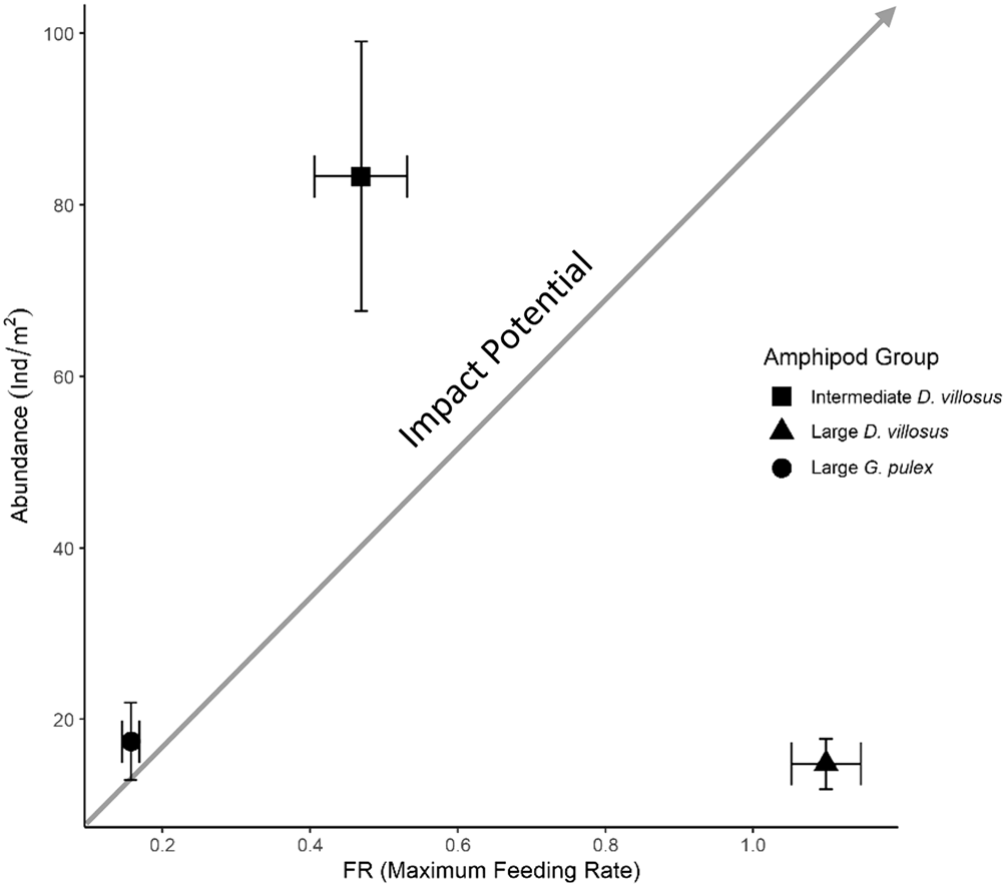
RIP biplots comparing intermediate *D. villosus* (filled square), large *D. villosus* (filled triangle) and large *G. pulex* (open circle) when feeding upon native *R. temporaria* larvae as prey. Biplots generated using mean ± standard errors (SE) estimates for FRs (i.e. maximum feeding rates) and field abundances (ind/m^2^) recorded in each amphipod size treatment. Mean (± SE) FR parameters are generated from bootstrapped estimates (n = 30 bootstraps).

The RIP metric returned a substantially greater impact potential in invasive *D. villosus*, relative to native *G. pulex* (Table 3). The RIP metric indicated that considerably higher RIP scores estimated for large and intermediate *D. villosus*, compared to *G. pulex*, were driven by different biological characteristics. Despite the non-significant differences in FR parameters between size-matched amphipods (Table 1), significantly superior field abundance estimates recorded for intermediate *D. villosus* generated a greater RIP score, than large *G. pulex* (Table 3). The RIP biplot illustrates this, with differential field abundance estimates generating a substantial vertical shift for intermediate *D. villosus* in comparison with the RIP for large *G. pulex* (Fig. 3). Large *D. villosus* displayed similar field abundances, when compared to large *G. pulex* (Table 3). However, significantly lower handling times, and subsequently higher maximum feeding rates, resulted in a superior RIP scores, with the RIP biplot highlighting a substantial shift to the right when compared to *G. pulex* (Fig. 3).

## Discussion

Over the past 20 years, *D. villosus* has spread rapidly throughout Europe^15^. Within invaded communities, *D. villosus* has a significant ecological impact, with considerable declines in native macroinvertebrate populations and altered ecosystem functioning ascribed to its competitive and predatory capabilities^14, 15, 42–46^. Whilst previous evidence concerning the predation of early stage amphibians by amphipods is scarce (see Fries & Tesch^47^; Hudgens & Harbert^48^), we provide the first empirical evidence that amphipods can kill and consume both embryonic and larval amphibians. Greater *per capita* feedings rates combined with higher densities in the field lead us to predict that invasive *D. villosus* might also exert a population-level effect upon amphibians within invaded communities.

Predation of invasive *X. laevis* embryos was observed, with *D. villosus* consuming a significantly larger number of embryos – increasing with invader body size—whilst predation by native *G. pulex* was negligible. When presented with native *R. temporaria* embryos, both native and invasive amphipods appeared incapable of consuming these larger embryos, although there was evidence of attempted predation. Predation of native *R. temporaria* embryos by invasive amphipods was only observed when embryos were damaged prior to exposure. *Rana temporaria* embryos are surrounded by a comparatively thick vitelline jelly capsule^49^, which protects the eggs from some predators^50^. *Rana temporaria* embryos may be susceptible to predators with piercing, sucking mouthparts, yet reasonable invulnerable to predators which possess chewing, biting mouthparts^51^, such as amphipods, thereby escaping potential predatory pressures exerted by invasive amphipods upon embryonic amphibians.

Invasive *X. laevis* embryos are comparably smaller than those generated by native *R. temporaria* (2.19 ± 0.02 mm and 7.83 ± 0.16 mm, respectively; pers. obs.), and other anurans^52^. Our findings suggest that *D. villosus* may present a predatory threat to other native amphibian species with relatively small embryos, such as the great crested newt (*Triturus cristatus*; embryo diameter =  ~ 4.5 mm), the smooth newt and the palmate newt (*Lissotriton vulgaris* and *L. helveticus*; embryo diameter =  ~ 3 mm^53^); of which several species have been recorded in regions invaded by *D. villosus* (e.g. Grafham Water and Pitsford Reservoir; Anglian Water, pers. comm; The Wildlife Trust for Bedfordshire, Cambridgeshire & Northamptonshire, pers. comm). Our findings also retain ecological relevance with regards to invasive *X. laevis* populations, given the previous existence of several feral populations in Great Britain^54^.

Native and invasive amphipods readily preyed upon *R. temporaria* larvae. Large *D. villosus* expressed significantly lower handling times, consuming early-stage amphibians at a greater rate than smaller amphipods, which translated into substantially higher maximum feeding rates, seven times greater than large *G. pulex*. As such, the higher *per capita* prey intake observed in significantly larger invaders is likely explained by the naturally larger body size of *D. villosus*, rather than any interspecific differences in innate predatory ability^16, 24^. Our observation of higher consumption by larger amphipods is consistent with previous studies^16, 24^, and by extension, general biological theory^55^. Metabolic theory predicts that metabolic rate typically exhibits positive allometric scaling relative to size^56^, with greater metabolic demand requiring high resource intake to maintain fundamental biological processes^57^.

Superior consumptions rates by larger amphipods may be facilitated by larger mouthparts and gnathopods, allowing individuals to capture and consume a wide range of prey^58^, or a larger gut capacity required to efficiently digest food items^59^. The presence of large glycogen reserves in *D. villosus*, compared to other amphipod species, allow this invader to overcome various anti-predatory evasion behaviours demonstrated by larval amphibians, such as burst-swimming^60^; a trait which appears superior in ranids, compared to other anurans (e.g. bufonids^61^). Given that *R. temporaria* larvae remain vulnerable to predation until complete metamorphosis^62^, we posit that the potential impact of invasive *D. villosus* could persist throughout development, with prolonged predation on growing larvae in the field continuing until they achieve a size or developmental stage which is invulnerable to *D. villosus*.

The comparative FR approach revealed that, when compared to native *G. pulex*, invasive *D. villosus* generally exhibited a higher Type II FR. This differential predatory response became more apparent in larger invaders, with significantly higher FRs exhibited by large *D. villosus* feeding upon embryonic and larval amphibians. Separation between FR curves generated for large invaders and size-matched amphipods across both prey systems would imply the potential for *D. villosus* to impose a greater predatory impact upon native amphibian populations, compared to native *G. pulex*.

Type II FRs are indicative of potentially unstable predator–prey interactions^63^. At higher prey densities, *per capita* predation rates decelerates to an asymptote as consumption become limited by consumer handling times^63^. As a result, an unstable equilibrium is attained, centred on the asymptotic point^63^. If predation levels exceed the growth capacity of prey populations existing at densities below the established equilibrium point, predator–prey dynamics may destabilise resulting in the extirpation of affected prey populations^63, 64^. Differential Type II FRs identified in comparative laboratory-based studies of predation on macroinvertebrate prey are consistent with observed field patterns of reduced macroinvertebrate diversity and abundance^15, 43^. Our findings suggest a similar threat may extend to amphibians in the field.

When compared to native amphipods, *D. villosus* exhibits notably higher fecundity and a short interbrood interval^18^, allowing this invader to rapidly form “super-abundant” populations in invaded regions^14^. The RIP metric highlighted a significantly greater impact potential by invasive *D. villosus* than by native *G. pulex*, driven by both larger body size and greater abundance of this invasive species. Large *D. villosus* exhibited a higher *per capita* impact than native *G. pulex*, translating into an RIP score 6.4 times stronger than native *G. pulex*. A superior RIP score was also predicted for intermediate *D. villosus*, when compared to *G. pulex*. Whilst the comparative FR approach identified no significant difference between *per capita* effects recorded in size-matched native and invasive amphipods, inclusion of field abundance estimates into the RIP metric detected a substantially higher impact potential for intermediate *D. villosus*, with significantly higher field densities (4.8 times higher than *G. pulex*) generating a predicted impact score approximately 15 times greater than its native counterpart.

The RIP metric considers the effect of differential field abundances of natives and invaders, but assumes that consumer interactions are explicitly advantageous^8^. In reality, interactions between consumers may be additive^65^, synergistic^66^, or antagonistic^67^. By incorporating such context dependencies into FR models, we might further refine predictions. Nevertheless, the RIP metric has proven to be an effective predictive tool when applied to previous literature. Estimations of invader RIP support alternative impact measurements (e.g. Laverty et al.^68^) and correspond to observed field impacts^8^. As such, the RIP metric has formed the foundation for several alternative quantitative metrics (see Dickey et al.^69^).

In the current study, the RIP metric highlighted a significantly greater impact potential by invasive *D. villosus* towards native early-stage amphibians, when compared to native *G. pulex*. These findings are consistent with similar magnitudinal patterns of differential impacts identified in *D. villosus* towards other freshwater organisms (see Dick et al.^8^). However, our estimates for the abundance of *D. villosus* in Grafham Water Reservoir were considerably lower than those recorded in other European and UK localities in which *R. temporaria* have been reported. The potential for *D. villosus* to reach higher densities indicate that the potential impact of this invader upon early-stage amphibians may be even stronger in other invaded regions.

## Conclusions

This is the first empirical evidence of predation of early-stage amphibians by freshwater amphipods. The invasive *D. villosus* exhibited consistently higher *per capita* predation rates upon invasive amphibian embryos and native amphibian larvae, with predation increasing as a function of invader body size. The detection of Type II FRs, significantly higher in large-bodied invaders, are indicative of the potential ecological impact of *D. villosus*, with higher predation rates predictive of a depletive, potentially destabilising effect upon amphibian populations, through the consumption of vulnerable embryos and larvae. This higher ecological impact, predicted for invasive *D. villosus*, is further intensified when the higher field abundances of this invasive amphipod are considered.

Large-bodied invasive predators are acknowledged as primary drivers of global amphibian declines^33, 34^. With evidence of potential co-occurrence between *D. villosus* and native amphibians, recorded in both UK freshwaters (Anglian Water, pers. comm.; Cardiff Harbour Authority, pers. comm.) and in mainland Europe^36–40^, the findings of the current study predict that the highly predacious ‘killer shrimp’ may further contribute to declining amphibian populations through the predation of early life-stages. Given the projected expansion of *D. villosus* in British freshwaters^70, 71^, we might predict the introduction of *D. villosus* into amphibian-rich areas in the near future, with consequences for amphibian populations expected to follow. However, further research is required to determine the suitability of different freshwater habitats which are typically used by breeding amphibians.

## Materials and methods

We compared the predatory impacts of invasive *D. villosus*, and the British-native amphipod, *G. pulex*, upon the early life-stages of amphibians. Initial experiments used invasive *X. laevis* embryos as a focal prey organism and established the potential for native and invasive amphipods to predate upon early-stage anurans. Therefore, experiments proceeded utilising the embryonic and larval forms of native *R. temporaria* as focal prey types. *Rana temporaria* have been recorded occupying the same habitat as *D. villosus*, both in UK invaded sites^36^ (The Wildlife Trust for Bedfordshire, Cambridgeshire & Northamptonshire, pers. comm), and in mainland Europe^37, 38^.

We compared size-matched amphipods to examine intrinsic differences between species, as well as significantly larger *D. villosus* to reflect natural differences in amphipod size^16, 24^. A comparative functional response (FR) approach was utilised to quantify amphipod predation upon invasive and native amphibian embryos and larvae. FRs are a fundamental measure of resource use frequently applied in invasion ecological research as a metric to assess trophic interactions; quantifying the relationship between *per capita* predation rate and prey abundance (i.e. FR). By comparing FRs of IAS and native analogues predictions can be made as to how differential predator behaviours might impact upon prey populations in the field^7, 8^.

When considering the absolute ecological impact of invasive predators, total invader impact should consider predatory capability, relative to native analogues, as well as relative abundance^8^. Based on the classic total response equation (Total Response = Functional Response x Numerical Response), the Relative Impact Potential (RIP) metric has recently been developed and incorporates relative consumer abundance or biomass—a proxy for numerical response—as a means of scaling relative *per capita* effects (i.e. FR) to predict the relative impact of an invasive predator in comparison with a native analogue (RIP = FR x Abundance^8^).

### Experimental organisms

Ethical consent was obtained from Natural England, the Home Office and the University of Leeds Ethics Committee. The use of freshly hatched, pre-feeding *R. temporaria* larvae for experimentation fell outside the remit of the Wildlife and Countryside Act 1981 (section 9.5; protected against sale only), and the UK Animal Scientific Procedures Act 1986 (ASPA; section 1.4.2). Animals were maintained in compliance with guidelines stated in the Code of Practice for the Housing and Care of Animals Bred, Supplied or Used for Scientific Purposes (section 3, chapter 11). All experimental work was conducted in accordance with relevant guidelines and regulations, including the maintenance, use and termination of study organisms.

*Xenopus laevis* embryos were sourced from adult females, commercially reared by the European Xenopus Resource Centre (EXRC, University of Portsmouth). In December 2016, embryos were transported to the laboratory, stored in isotonic 1 × Modified Barth’s Saline (MBS) solution (Supplementary Materials Table S1). Upon receipt, embryos were gradually transferred into aerated dechlorinated tap water over the course of several hours and kept at 14.0 ± 0.1 °C under a 12:12 h light:dark regime, as recommended by the EXRC.

Freshly deposited native *R. temporaria* embryos (approximately 36 h post-fertilisation) were collected between February and March 2017 from several freshwater sites around West Yorkshire (Supplementary Materials Table S2). Embryos were removed as whole clutches and approximately halved, with half of the clutch transferred to 2L sterile storage containers with site-sourced water, and half of the clutch returned to the site. Harvested embryos accounted for less than 10% of the total population of embryos present at each site.

Embryos were transported to the laboratory in insulated boxes and stored as individual half-clutches in aerated aquaria with dechlorinated tap water at 4.0 ± 0.2 °C under a 12:12 h light:dark regime. By maintaining the embryos at 4 °C, the rate of embryonic development was reduced considerably (~ 30 days to hatching), maximising the potential experimental period whilst enabling greater control over developmental progress.

To obtain *R. temporaria* larvae for experimentation, embryos were reared to early-stage larvae. When larvae, still encapsulated in vitelline jelly, began to develop external gill filaments and exhibited neuromuscular reflex responses (i.e. Gosner stage or G 18–19; see Gosner^72^), they were transferred to 14 °C (consistent with ambient temperatures recorded during field sampling) in preparation for hatching. Transference to the higher temperature regime, conducted gradually over the course of 24 h, accelerated development, with hatching typically occurring within approximately 24 h of changing temperature regimes. Shifting temperature regimes also allowed larvae to acclimatise to warmer conditions prior to experimentation. Conditions were sufficient to produce high rates of larval hatching (> 75%) and survival (~ 70%). Embryonic and larval stock tanks were cleaned twice weekly. Only recently hatched, pre-feeding larvae (i.e. G. 19–20^72^), lacking any obvious functioning mouthparts and relying solely on the yolk sac for nutrition^73^, were used for experimentation.

### Amphipods

Invasive *D. villosus* were sampled from artificial substrates in Grafham Water, Cambridgeshire (52°17′31.2′′N 0°19′23.6′′W), and native *G. pulex* were kick-sampled from Meanwood Beck, West Yorkshire (53°49′49.2′′N 1°34′31.3′′W). Amphipod species were identified based on urosome morphology^74, 75^. Each species was independently maintained in the laboratory in 4L aquaria with aerated, dechlorinated tap water and provided an ad libitum diet of stream-conditioned sycamore leaves (*Acer pseudoplatanus* L.), conditioned for approximately one month. Amphipod specimens were maintained at 14.0 ± 0.1 °C under a 12:12 h light:dark regime for at least 96 h before experimental use.

Male amphipods were used in experimental treatments as females may exhibit variations in predatory behaviour^76^. Male *G. pulex* were identified via precopulatory pairings, whilst male *D. villosus* were identified by the presence of genital papillae, and the absence of oostegites (i.e. brood plates). Amphipods exhibiting visible parasitic infections were excluded from experimentation, controlling for potential variations in behaviour caused by infections^77, 78^. Amphipods were kept in sex-specific communal tanks for at least 24 h prior to their use in experimental trials, and were only used once in each experimental treatment.

Given the significantly larger natural body size of *D. villosus* when compared to other European gammaridean amphipods^21^, amphipods were categorised into three size groups; large *G. pulex*, intermediate *D. villosus* and large *D. villosus*. Controlling for amphipod body size enabled fundamental comparisons of inherent differences in predatory impact between size-matched native (large *G. pulex*) and invasive (intermediate *D. villosus*) amphipod groups, whilst also considering the predicted amplificatory effect that larger natural body size in *D. villosus* may have on maximal predatory impact^16^.

Amphipods were blotted dry, weighed and photographed in a resting curved state, with measurements taken approximately 2 h prior to the starvation of amphipods in preparation of experimental trials. Body length was measured as a curved line from the rostrum tip to urosome base in Image J (http://rsbweb.nih.gov/ij/). Rarefaction of datasets using size parameters recorded for amphipod groups used across all experimental systems indicated appropriate size-matching between large *G. pulex* (mean ± standard error (SE), length = 16.356 ± 0.121 mm; weight = 57.461 ± 0.779 mg) and intermediate *D. villosus* (length = 16.656 ± 0.132 mm; weight = 57.314 ± 0.856 mg; p > 0.05 for both body parameters). Large *D. villosus* were significantly larger (23.481 ± 0.130 mm) and heavier (146.218 ± 1.963 mg) than size-matched *D. villosus* and *G. pulex* in both experiments (p < 0.001 for both; see Supplementary Materials Table S3).

### Functional response (FR) experiments

#### Experimental design

To compare predatory FRs of native and invasive amphipods against amphibian prey, three independent experiments were conducted in which amphipods were presented with amphibian prey in varying densities. The first experiment compared FRs between native and invasive amphipods towards invasive *X. laevis* embryos. The second experiment compared amphipod FRs towards native *R. temporaria* embryos. The third experiment assessed amphipod FRs towards *R. temporaria* larvae.

Prior to experimentation, individual amphipods were placed in clear plastic arenas (90 mm diameter, 50 mm height) with 250 ml of dechlorinated tap water, and starved for 24 h. A single glass bead (20 mm diameter, 9 mm height) was placed in arenas as substrate, providing amphipods with shelter and to prevent continuous swimming. Amphipods were then transferred to experimental arenas, identical to those described above, containing a known number of invasive *X. laevis* embryos (2, 4, 6, 8, 10, 15, 20, 40 or 70 embryos), native *R. temporaria* embryos (1, 2, 3, 5, 8, 10 or 15 embryos) or freshly hatched native *R. temporaria* larvae (1, 2, 3, 5, 8, 10 or 15 tadpoles; Supplementary Materials Table S4). Prey were situated in arenas two hours prior to the introduction of amphipod predators and the commencement of trials.

Experimental trials began with the introduction of a single amphipod predator and were conducted at 14 ± 0.1 °C under a 12:12 h light:dark regime. Trials continued for 24 h (*X. laevis*/*R. temporaria* embryos) or 48 h (*R. temporaria* larvae), without replacing consumed prey. Trials concluded with the removal of amphipod predators and the enumeration of alive, dead or consumed prey. Dead prey which did not exhibit signs of predation were assumed to reflect background mortality (< 1.24% in all experiments). After terminating experimental trials, amphipods were monitored for a further 24 h. Amphipods that moulted or died were excluded from analysis. Following rarefaction to ensure appropriate size-matching, data pertaining to embryonic prey treatments was retained for five replicates, whilst the larval prey treatment comprised up to eleven replicates at all prey densities. Controls consisted of five (embryos) or eleven (larvae) replicates of each prey density, without amphipod predators present.

#### Field sampling: estimating amphipod abundance

In November 2017, field sampling was undertaken at several un/invaded freshwater sites within Great Britain to estimate field abundances of native *G. pulex* and invasive *D. villosus* within these regions. *Dikerogammarus villosus* were sampled from six sites situated around the perimeter of Grafham Water, Cambridgeshire (Supplementary Materials Table S5). Sampling was conducted approximately 2 m from the shoreline within a 50 × 50 cm area (0.25m^2^), using a modified, bottomless receptacle (50 cm diameter, 65 cm height, 80L volume) which allowed access to the underlying substrate. Over a five-minute period, the substrate was agitated and netted, followed by two minutes of netting through the water column.

*Gammarus pulex* were sampled from five sites along Adel Beck and Meanwood Beck, West Yorkshire (Supplementary Materials S5). Sampling was conducted in the centre of these lotic systems, within a 50 × 50 cm area (0.25 m^2^). The substratum was agitated for 5 min and any amphipods dislodged were collected in a surber sampler. A further two minutes were spent hand sampling larger rocks present within the sampling area.

Amphipod specimens were stored in 70% ethanol. In the laboratory, amphipod specimens were sorted into size categories, matching those selected for FR trials, and enumerated.

Abundance data was supplemented using estimates reported in previously published literature; recorded for *G. pulex* within native ranges^78–85^, and *D. villosus* within invaded ranges^39, 81, 84, 85–93^. Data was taken from studies of amphipod abundance at sites where the presence of native *R. temporaria*, and other European amphibian species, had also been recorded (amphibian occurrence taken from recording databases^36, 94, 95^). Using published abundance estimations, the number of large *G. pulex*, intermediate *D. villosus* and large *D. villosus* was calculated, based on the proportional abundance of each amphipod size group recorded during field sampling.

### Statistical analysis

Statistical analyses were performed in R studio, version 3.3.2^96^. FR analyses were conducted using an integrated package for functional response analysis in R (*frair*, version 0.5.100^97^).

### Functional response analysis

#### Phenomenological functional response analysis

A phenomenological approach was applied to each experimental combination (amphipod x prey type) to determine FR type (I, II, III) based on the general shape of the response curve. For each amphipod x prey type combination, logistic regressions of proportional prey consumption against prey density were performed, fitted with a quasibinomial error distribution to account for overdispersion. A significant negative first-order term was indicative of a Type II FR, whilst a significant positive first order term, superseded by a significant negative second order term denoted a Type III FR^64^.

#### Mechanistic functional response analysis

Where analyses suggested that Type II FRs were appropriate, FRs were modelled using the Rogers’ random predator equation (Eq. 1)^98^. This model accounts for the depletion and non-replacement of prey^64^.

1 $${N}_{e}= {N}_{O}\left(1-exp\left(a\left({N}_{e}h-T\right)\right)\right)$$

where ${N}_{e}$ is the number of prey consumed, ${N}_{O}$ is the initial density of prey, $a$ and $h$ represent the mechanistically explicable coefficients for attack coefficient ($a$) and handling time ($h$), and $T$ is the total experimental period in days.

Using these parameters, maximum feeding rate was calculated as $1/Th$. FR models were fitted using the *frair_fit* function, which utilises maximum likelihood estimations (*bbmle::mle2*, version 1.0.20^99^), and a modified version of Eq. (1), incorporating the *Lambert W* function to resolve the presence of ${N}_{e}$ on either side of the equation (Eq. 2).

2 $${N}_{e}= {N}_{O}-\frac{lambertW\left(a\bullet h\bullet {N}_{O}\bullet exp\left(-a\bullet \left(T-h\bullet {N}_{O}\right)\right)\right)}{(a\bullet h)}$$

Comparisons of attack coefficient ($a$) and handling time ($h$) were conducted between amphipod groups (within each prey type) using an ‘indicator variable’ approach to explicitly model differences in the parameter estimates for each amphipod group (*frair_compare* function; see Juliano^64^, Pritchard et al.^97^, Taylor & Dunn^24^).

Each fitted FR model was non-parametrically bootstrapped (n = 2000) to generate 95% confidence intervals, thereby visualising model variability. Additional non-parametric bootstrapping (n = 30) was applied to models, allowing multiple estimates of handling time ($h$), and thus maximum feeding rate ($1/Th$) to be calculated^16, 100, 101^. This generated mean (± SE) estimated maximum feeding rates for RIP calculations.

In FR experiments which utilised invasive *X. laevis* embryos as focal prey, negligible predation recorded in native *G. pulex* prevented comparisons of FR curves and parameters between native and invasive amphipods. Instead, Chi-square (χ^2^) tests were applied to compare the frequency of predation (i.e. proportion of individuals that consumed embryos) recorded between amphipods. Chi-squared tests were also conducted for FR experiments with native *R. temporaria* larvae, given the relatively low incidence of predation recorded in size-matched amphipod groups, compared to large *D. villosus*. In FR experiments with native *R. temporaria* embryos, a complete absence of predation prevented statistical analyses.

#### Amphipod field abundance estimates

Field abundance estimates for native and invasive amphipods were compared using a generalised linear model (GLM), fitted with a quasipoisson error distribution to account for overdispersion. A *post-hoc* Tukey HSD test (α = 0.05; *multcomp::glht*, version 1.4–8^102^), was subsequently conducted to compare field abundance estimations between amphipods.

#### Relative impact potential (RIP) analysis

Mean (± SE) estimates for maximum feeding rates (i.e. FR), generated from bootstrapped models, and field abundances were incorporated into the RIP metric, enabling pairwise comparisons of relative impact potential between invasive and native amphipods. This allowed RIP probabilities and confidence intervals to be generated for invasive amphipods, when compared to native *G. pulex*. Due to the potential ecological significance of invader predation upon native amphibian species, RIP analyses focussed on FR models pertaining to the predation of native *R. temporaria* larvae. ‘RIP biplots’ were generated, presenting the RIP values of the three amphipod groups using field abundance estimates as a proxy for numerical response (see Laverty et al.^100^; Cuthbert et al.^101^).

## Supplementary Information

Supplementary Information.

Supplementary Information.

Supplementary Information.

## Data Availability

Raw data pertaining to functional response analyses are available in the electronic supplementary material.
