# Supplementary material for: Superior predatory ability and abundance predicts potential ecological impact towards early-stage anurans by invasive ‘Killer Shrimp’ (Dikerogammarus villosus)
Source: Sci Rep. 2021 Feb 25;11:4570. doi: 10.1038/s41598-021-82630-5 (PMC7907340; doi:10.1038/s41598-021-82630-5)
Supplement: Supplementary file 3 — Supplementary Information. [file 41598_2021_82630_MOESM3_ESM.docx]

**Supplementary Materials**

for Warren, D.A., Bradbeer, S.J. & Dunn, A.M.
Amphibians at Risk: Superior Predatory Ability and Abundance Predicts Significant Ecological Impact Towards Early-Stage Anurans by Invasive Killer Shrimp (*Dikerogammarus villosus*)

Table S1 – List of reactants and their quantities used to make 1 x Modified Barth’s Saline (MBS) solution (pH 7.8) up to a volume of 1 L.

| Reagent | Quantity (g) | Final Concentration (mM) |
| --- | --- | --- |
| NaCl | 5.143 | 88 |
| KCl | 0.075 | 1 |
| MgSO_4_ | 0.120 | 1 |
| HEPES | 1.192 | 5 |
| NaHCO_3_ | 0.210 | 2.5 |
| CaCl_2_, dihydrate | 0.103 | 0.7 |
| Deionised Water | to 1 L |  |

Note: Adjust pH of final solution to 7.8 by adding 10 M of NaOH and autoclave solution to sterilise.
[Accessed from: <http://cshprotocols.cshlp.org/content/2009/9/pdb.rec11946.full?text_only=true>]

Table S2 – List of freshwater field sites situated within West Yorkshire which were sampled for freshly deposited native *Rana temporaria* embryos.

| Field Site | Coordinates |
| --- | --- |
| Nell Bank Environmental Study Centre, Ilkley | lat 53^o^56’00.2"N, long 1^o^48’26.0"W |
| Meanwood Park, Leeds | lat 53^o^50’23.0"N, long 1^o^34’34.8"W |
| Middleton Park, Leeds | lat 53^o^45’15.4"N, long 1^o^32’49.0"W |
| Farnley Hall Fishpond Local Nature Reserve, Leeds | lat 53^o^47’17.3"N, long 1^o^37’23.2"W |

Table S3 – The mean (+ SE) length and weight (i.e. body size) of amphipod groups used in each experimental system. Prior to experimentation, live amphipods were blotted dry before weight (mg) was measured; lengths (mm), taken from the tip of the rostrum to the base of the urosome for amphipods in curved, resting states, were measured from digital photographs. Across both experimental systems, ‘large’ *D. villosus* were significantly larger and heavier than both ‘intermediate’ *D. villosus* and ‘large’ *G. pulex*, which in turn did not significantly differ in size.

| Prey Treatment | Body parameter | ‘Large’ *D. villosus* | | ‘Intermediate’ *D. villosus* | | ‘Large’ *G. pulex* | |
| --- | --- | --- | --- | --- | --- | --- | --- |
|  |  | Mean | SE | Mean | SE | Mean | SE |
| *Xenopus laevis* embryos | Length | 22.04 | 0.29 | 14.36 | 0.12 | 14.36 | 0.13 |
|  | Weight | 165.03 | 3.21 | 44.31 | 1.09 | 46.49 | 1.34 |
| *Rana temporaria* embryos | Length | 23.85 | 0.23 | 17.49 | 0.17 | 16.44 | 0.15 |
|  | Weight | 146.78 | 4.10 | 62.18 | 1.29 | 62.67 | 1.26 |
| *Rana temporaria* larvae | Length | 23.97 | 0.13 | 17.32 | 0.12 | 17.23 | 0.11 |
|  | Weight | 137.41 | 2.34 | 61.01 | 0.88 | 60.12 | 0.74 |

Note: Length and weight data recorded for each amphipod group was compiled into a single dataset for all three experimental treatments (i.e. *X. laevis* embryos, *R. temporaria* embryos and *R. temporaria* larvae). Length and weight data was analysed using a non-parametric Kruskal-Wallis test, due to non-normal residuals even following log-transformation. Pairwise *post-hoc* comparisons were conducted using the non-parametric Dunn’s test, with Bonferroni adjusted p-values (*PMCMRplus::kwAllPairsDunnTest*, version 1.4.2; Pohlert, 2015).
Non-parametric analysis indicated a statistically significant difference in the length (χ^2^ = 293.60, df = 2, p < 0.001) and weight (χ^2^ = 293.02, df = 2, p < 0.001) of the three amphipod size groups. *Post-hoc* tests confirmed that across all experimental treatments, ‘large’ *D. villosus* were significantly longer and heavier than both ‘intermediate’ *D. villosus* and *G. pulex* (Dunn test adjusted p < 0.001 for both tests). *Gammarus pulex* and ‘intermediate’ *D. villosus* did not differ in length or weight (Dunn test adjusted p > 0.05 for both tests).

Table S4 – Length (+ SE) and approximate developmental stage of amphibian embryos and larvae used in experiments. n = 30, except for *R. temporaria* larvae n = 27. As amphibian embryos develop, vitelline jelly thickness varies ^2^. Therefore, only embryos with complete vitelline membranes that were robust to manipulation were used to standardise prey life-stages. Similarly, throughout larval development, larval swimming capability also alters ^3^. As such, only larvae between 12 h and 24 h post-hatching were selected.

| Prey Type | Stage | Length (mm) | SE |
| --- | --- | --- | --- |
| *X. laevis* embryos | NF 10* | 2.19 | 0.02 |
| *R. temporaria* embryos | G 10** | 7.83 | 0.16 |
| *R. temporaria* larvae | G 20** | 14.82 | 0.31 |

Length of embryos is diameter, including the vitelline jelly capsule. Length of larvae is taken from the anterior tip of the head/body to the posterior tip of the tail.
*R. temporaria* larvae were measured after killing in 70% ethanol
Note: NF = Nieuwkoop and Faber Stage ^4^, G = Gosner Stage ^5^.

Table S5 – List of British field sites sampled to obtain estimates of population abundance for native *Gammarus pulex* and invasive *Dikerogammarus villosus*. These sites predominantly comprised of rocky substrate, which is frequently favoured by *D. villosus* and *G. pulex* as suitable habitat ^6,7^.

| Amphipod Species | Field Site | County | Coordinates |
| --- | --- | --- | --- |
| *G. pulex* | Golden Acres Park | West Yorkshire | lat 53^o^52’07.3" N, long 1^o^35’19.1" W |
|  | Adel Woods | West Yorkshire | lat 53^o^51’33.0" N, long 1^o^34’43.9" W |
|  | Meanwood Valley Trail | West Yorkshire | lat 53°50'36.9" N, long 1°34'11.1" W |
|  | Meanwood Park (Upper) | West Yorkshire | lat 53°50'23.8" N, long 1°34'35.3" W |
|  | Meanwood Park (Lower) | West Yorkshire | lat 53°49'49.2" N, long 1°34'31.3" W |
| *D. villosus* | Valley Creek | Cambridgeshire | lat 52°17'26.8" N, long 0°19'43.7" W |
|  | Grafham Water Fishing Lodge | Cambridgeshire | lat 52°17'28.8" N, long 0°19'28.1" W |
|  | Gaynes Cove | Cambridgeshire | lat 52°17'07.3" N, long 0°17'44.0" W |
|  | Grafham Water Visitors Centre | Cambridgeshire | lat 52°17'51.1" N, long 0°17'34.6" W |
|  | Hedge End | Cambridgeshire | lat 52°18'18.2" N, long 0°18'15.6" W |
|  | Hill Farm Bay | Cambridgeshire | lat 52°18'36.9" N, long 0°19'08.0" W |

Table S6 – Results of logistic regression of the proportion of prey consumed in each prey density treatment for each amphipod size and prey treatment. Analyses were conducted using a quasi-binomial error structure. A statistically significant negative first order term is indicative of a Type II FR ^8^.

| Prey treatment | Amphipod group | First Order Term – Estimate (+SE) | z | P |
| --- | --- | --- | --- | --- |
| *X. laevis* embryos | ‘Intermediate’ *D. villosus* | -0.041 (+0.004) | -8.547 | < 0.001*** |
|  | ‘Large’ *D. villosus* | -0.039 (+0.004) | -9.634 | < 0.001*** |
| *R. temporaria* larvae | ‘Large’ *G. pulex* | -0.143 (+0.059) | -2.428 | 0.018* |
|  | ‘Intermediate’ *D. villosus* | -0.121 (+0.044) | -2.727 | < 0.01** |
|  | ‘Large’ *D. villosus* | -0.112 (+0.028) | -3.967 | < 0.001*** |

Asterisks indicate significance of P values; * = P < 0.05, ** = P < 0.01, and *** = P < 0.001.

References

1. Pohlert, T. *PMCMR: Calculate Pairwise Multiple Comparisons of Mean Rank Sums (Version 4.0)*. (2015).

2. Roberts, R. *The Frog: Its Reproduction and Development*. (The Blakiston Company, 1951).

3. Van Buskirk, J. & McCollum, S. a. Influence of tail shape on tadpole swimming performance. *J. Exp. Biol.* **203**, 2149–2158 (2000).

4. Nieuwkoop, P. D. & Faber, J. *Normal Table of Xenopus Laevis (Daudin): A Systematical & Chronological Survey of the Development from the Fertilized Egg till the End of Metamorphosis*. (Garland Publishing, 1994).

5. Gosner, K. L. A Simplified Table for Staging Anuran Embryos Larvae. *Herpetodologists’ Leag.* **16**, 183–190 (1960).

6. Elliott, J. M. Day-night changes in the spatial distribution and habitat preferences of freshwater shrimps, Gammarus pulex, in a stony stream. *Freshw. Biol.* **50**, 552–566 (2005).

7. MacNeil, C. *et al.* The Ponto-Caspian ‘killer shrimp’, Dikerogammarus villosus (Sowinsky, 1894), invades the British Isles. *Aquat. Invasions* **5**, 441–445 (2010).

8. Juliano, S. A. Nonlinear Curve Fitting: Predation and Functional Response Curves. in *Design and Analysis of Ecological Experiments* (eds. Cheiner, S. M. & Gurven, J.) 178–196 (Chapman and Hall, 2001).
